# Cotinine inhibits the pro-inflammatory response initiated by multiple cell surface Toll-like receptors in monocytic THP cells

**DOI:** 10.1186/1617-9625-10-18

**Published:** 2012-11-23

**Authors:** Juhi Bagaitkar, Iris Zeller, Diane E Renaud, David A Scott

**Affiliations:** 1 Microbiology and Immunology, University of Louisville, Louisville, KY, 40292, USA; 2 Oral Health and Systemic Disease Research Group, University of Louisville, Louisville, KY, 40292, USA; 3 Currently: Department of Pediatrics, Washington University School of Medicine, St. Louis, MO, USA

**Keywords:** Cholinergic anti-inflammatory pathway, Cotinine, Cytokines, GSK3β, Inflammation, THP-1 cells, Tobacco smoking, Toll-like receptors

## Abstract

**Background:**

The primary, stable metabolite of nicotine [(S)-3-(1-methyl-2-pyrrolidinyl) pyridine] in humans is cotinine [(S)-1-methyl-5-(3-pyridinyl)-2-pyrrolidinone]. We have previously shown that cotinine exposure induces convergence and amplification of the GSK3β-dependent PI3 kinase and cholinergic anti-inflammatory systems. The consequence is reduced pro-inflammatory cytokine secretion by human monocytes responding to bacteria or LPS, a TLR4 agonist.

**Findings:**

Here we show that cotinine-induced inflammatory suppression may not be restricted to individual Toll-like receptors (TLRs). Indeed, in monocytic cells, cotinine suppresses the cytokine production that is normally resultant upon agonist-specific engagement of all of the major surface exposed TLRs (TLR 2/1; 2/6; 4 and 5), although the degree of suppression varies by TLR.

**Conclusions:**

These results provide further mechanistic insight into the increased susceptibility to multiple bacterial infections known to occur in smokers. They also establish THP-1 cells as a potentially suitable model with which to study the influence of tobacco components and metabolites on TLR-initiated inflammatory events.

## Findings

### Introduction

The ability to regulate against prolonged or excessive inflammation is critical in preventing the onset of septic shock and the host-mediated damage associated with multiple chronic inflammatory diseases. On the other hand, a reduced inflammatory capacity can lead to an inability to clear pathogens and can compromise tissue remodeling [1].

In recent years the importance of the cholinergic anti-inflammatory system in modulating cytokine production in response to inflammatory stimuli has become apparent. The cholinergic anti-inflammatory pathway suppresses inflammation through the activation of the α7 nicotinic acetylcholine receptor (α7nAChR) on innate immune cells by neuronal and / or locally produced acetylcholine [1-4].

Nicotine, a major component of cigarette smoke, is also a potent α7nAChR agonist [5,6]. Smokers are more susceptible to multiple bacterial diseases than non-smokers [7]. The inappropriate activation of the cholinergic anti-inflammatory pathway by nicotine provides mechanistic insight into this phenomenon. Nicotine is rapidly converted into multiple metabolites in humans. Of these, cotinine, a considerably more stable molecule than nicotine that frequently reaches concentrations in the blood of > 500 ng/ml in tobacco smokers, is the most common [8]. We have previously shown that cotinine dramatically alters the nature of the inflammatory response to Gram negative bacteria in human innate cells [6]. Cotinine abrogates the production of cytokines that are under the transcriptional control of the NFκB system (TNF-α, IL-1β, IL-6, IL-12/IL-23 p40) and shifts the response towards an IL-10-dominated, anti-inflammatory profile in primary human monocytes stimulated with *Porphyromonas gingivalis* or LPS. This anti-inflammatory phenomenon is initiated upon engagement of leukocytic α7 nAChRs and is PI3K/GSK-3β-dependent but NFκB-independent [6].

Innate cells express multiple TLRs that recognize variant microbial-associated molecular patterns and induce the inflammatory response to pathogens, which is reflected in an increased secretion of pro-inflammatory cytokines. While cotinine represses the cytokine response to intact Gram negative bacteria, the specific TLRs whose cytokine output is manipulated by cotinine have yet to be established. Since its introduction in 1980 [9], the human monocytic-derived THP-1 cell line has been routinely used to study the functions of mononuclear innate cells *in vitro*. THP-1 cells are capable of further differentiation into macrophage-, foam cell- and osteoclast-like cells [10,11]. THP-1 cells express all surface-exposed TLR subunits (1, 2, 4, 5 and 6) [12,13] but do not have the genetic variation in TLRs noted in primary innate cells [14]. Furthermore, THP-1 cells are responsive to inflammatory stimuli [10,13]. Thus, THP-1 cells may represent a suitable model with which to study the effects of tobacco alkaloids and other anti-inflammatory agents that act via nicotinic receptors to subvert TLR signaling events. As the specific TLRs whose cytokine output is manipulated by cotinine have yet to be established and because we wished to see if THP-1 may be a useful model with which to study tobacco alkaloid-mediated immune suppression, the influence of cotinine on cytokine secretion upon stimulation of multiple TLRs was determined in THP-1 cells. Initially, we have focused on the surface exposed TLRs, rather than nucleotide oligomerization domain-like receptors (NLRs), retinoic acid-inducible protein 1-like receptors (RLRs) and cytosolic TLRs.

## Methods

### Growth and maintenance of THP-1 cells

THP-1 cells (Invivogen*,* San Diego, CA) were cultured in RPMI 1640 medium supplemented with 10% heat inactivated FBS, 50 μM 2-mercaptoethanol, 1 mM sodium pyruvate, 2 mM L-glutamine, 20 mM HEPES, 50 U/ml of penicillin and 50 μg/ml of streptomycin and maintained at 37°C, 5% CO_2_.

### Influence of cotinine on TLR-specific cytokine output

0.5 x 10^6^ THP-1 cells per well were seeded in 96-well plates and stimulated with human TLR-specific agonists (Pam3CSK4 [TLR2/1]; FSL-1 [Pam2CGDPKHPKSF, TLR2/6); *Escherichia coli K12* LPS [TLR4]; and *Salmonella typhimurium* flagellin [TLR5]*;* all Invivogen) at 1 μg/ml with or without a 2 hour pre-incubation with cotinine (10–1000 ng/ml; Sigma-Aldrich, St. Louis, MO). TNF levels were determined in 24 hr supernatants by ELISA (eBioscience, San Diego, CA).

### Statistical approaches

Statistical significance between groups was evaluated by ANOVA using the InStat program (GraphPad Software, San Diego, CA). Differences between groups were considered significant at the level of *p* < 0.05.

## Results and discussion

The ability of cotinine to suppress TNF secretion on engagement of TLR2/1, -2/6, -4 and −5 by TLR-specific cognizant ligands was determined. Concentrations of TNF secreted into 24 hour cell-free supernatants in unstimulated cells (mean, *s.d.* 495, *79* pg/ml) and those stimulated with 10, 100 and 1000 ng/ml cotinine alone (mean, *s.d.* 729, *157*; 744, *122*; and 838, *121* pg/ml, respectively) were minimal. Therefore, cotinine itself does not induce meaningful TNF release from monocytic cells.

**Figure 1:**
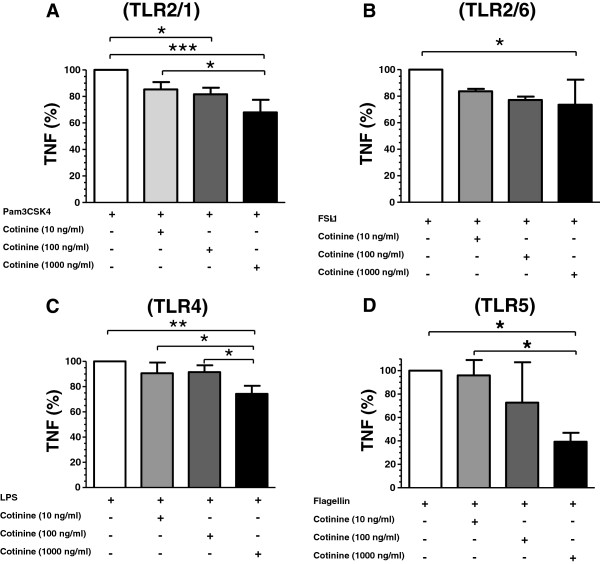
**Cotinine-induced suppression of cytokine induction on specific stimulation of TLRs.** TNF secretion was monitored following stimulation with TLR specific agonists at 1μg/ml (**A**, Pam2CGDPKHPKSF [TLR2/1]; **B**, FSL [TLR2/6); **C**, LPS [TLR4]; and **D**, flagellin [TLR5]) with and without cotinine pre-incubation (10–1000 ng/ml, 2 hr). Results are presented as percentage of TLR2/1, TLR2/6, TLR4 and TLR 5 agonist alone. 100% represents (mean ± s.d.) 25568 ± 1560 ng/ml, 10347 ± 2842 ng/ml, 4671 ± 117 ng/ml and 1583 ± 176 ng/ml for Pam2CGDPKHPKSF, FSL, LPS and flagellin, respectively. */**/*** ***p*** < 0.05, 0.01 and 0.001, respectively, compared to agonist only.

Cotinine suppressed TNF release in response to TLR2/1, TLR2/6, TLR4 and TLR5 agonists - Pam2CGDPKHPKSF (a synthetic diacylated lipoprotein); FSL (a synthetic triacylated lipoprotein), *E. coli* LPS and *S. typhimurium* flagellin, respectively – in a dose-related manner, as shown in Figure 1A-D. These results are consistent with the prior finding that cotinine suppresses pro-inflammatory cytokines and promotes IL-10 production in TLR4-stimulated primary human monocytes [6]. However, it should be noted that the degree of suppression varied by TLR and cytokine inhibition was less impressive than in primary cells. Also of interest is the observation by Noakes et al. that the ability of cord blood mononuclear cells to respond to TLR agonists is inversely proportional to the maternal smoking load [15]. Indeed, the cotinine doses employed herein are physiologically relevant to smokers (up to 1000 ng/ml, systemically) and those exposed to second-hand smoke (</= 10 ng/ml, systemically) [8]. Thus, this study furthers our understanding of how tobacco smoke exposure renders individuals more susceptible to bacterial infection by, e.g., *Streptococcus pneumonia*, *Neisseria meningitidis*, *Haemophilus influenza*, *Legionella pneumophila, Helicobacter pylori* and *Mycobacterium tuberculosis*[7].

It will be important, in the future, to perform similar studies in leukocytes from smoking and non-smoking humans. However, and more importantly, these findings are relevant to the expansive ongoing research aimed at exploitation of endogenous anti-inflammatory pathway(s) to either up-regulate or down-regulate the production of specific cytokine groups (pro- or anti-inflammatory cytokines) depending on the clinical necessity. Indeed, such therapeutic advances may be useful to both smokers and non-smokers, alike. To this end, THP-1 cells may represent a useful model with which to study anti-inflammatory agents that act via modulation of innate cell nicotinic receptor signaling pathways.

## Competing interests

DAS is the holder of U.S. Patent Application PCT/US2008/054569, *Therapeutic Cotinine Compositions*. Cotinine stimulates the cholinergic anti-inflammatory pathway which augments GSK3β anti-inflammatory events [6]. The authors have no other interests that might be perceived to influence the results and discussion reported in this paper.

## Authors' contributions

JB carried out the THP-1 stimulations and ELISAs; was involved in data analysis and interpretation; and provided input into manuscript revision. IZ was involved in data interpretation and helped draft and revise the manuscript. DER grew and maintained the THP1 cells and provided input into manuscript revision. DAS contributed to the study conception and design; interpretation of data; and drafting of the manuscript. All authors read and approved the final manuscript.
